# An Inch Screw Swallowed by a Child Two Years Old

**Published:** 1876-08

**Authors:** J. D. Westervelt

**Affiliations:** Longview, Ga.


### AN INCH SCREW SWALLOWED BY A CHILD TWO YEARS OLD.

BY J. D. WESTERVELT, M.D.. LONGVIEW, GA.

A few days since I was called to see a child of Mr. and Mrs. S——, who had, a short time previously, swallowed a sharp-pointed inch screw. Mrs. S—— informed me that it lodged for a short time in the throat, choking the child nearly to death, and in attempting to drag it out with her finger, pushed it beyond the constriction.

The child was cheerful when I saw it; respiration and deglutition easy; solid food was allowed; in seventy-two hours the screw was passed off entirely encapsuled in the peel of a baked apple.

